# Occupational stress, job satisfaction, and intention to leave among critical care nurses during the COVID-19 pandemic: a cross-sectional study in Lebanon

**DOI:** 10.3389/fpubh.2026.1814217

**Published:** 2026-05-29

**Authors:** Manar Bani-Hani, Suhair Al-Ghabeesh, Ahmad Rayan, Patricia Tai, Mirna Fawaz, Zeina Salloum, Hanan Alyami

**Affiliations:** 1 Faculty of Nursing, Al-Zaytoonah University of Jordan, Amman, Jordan; 2 Faculty of Nursing, Zarqa University, Zarqa, Jordan; 3 Department of Oncology, University of Saskatchewan, Saskatoon, SK, Canada; 4 Faculty of Health Sciences, Nursing Department, Beirut Arab University, Beirut, Lebanon; 5 Department of Medical and Surgical Nursing, College of Nursing, Princess Nourah bint Abdulrahman University, Riyadh, Saudi Arabia

**Keywords:** critical care nurses, intent to leave, COVID-19, occupational stress, job satisfaction, public mental health

## Abstract

**Background:**

In the context of an ongoing nursing shortage and severe economic crisis in Lebanon, the COVID-19 pandemic placed significant strain on the Lebanese healthcare system, particularly affecting frontline nurses’ psychological well-being and work conditions.

**Purpose:**

This study aimed to examine the relationship between occupational stress, job satisfaction, and intention to leave among nurses caring for COVID-19 patients in Lebanon.

**Methods:**

A cross-sectional correlational design was conducted among a convenience sample of 112 critical care nurses working in a university hospital in Lebanon. Data were collected using a self-administered questionnaire that included the MERS-CoV Staff Survey and the McCloskey/Mueller Satisfaction Scale (MMSS).

**Results:**

Findings revealed high levels of occupational stress and moderate levels of job satisfaction among participants. More than half of the nurses reported an intention to leave their current position. A significant negative relationship was found between stress related to infection stigma and job satisfaction.

**Conclusion:**

These findings highlight the urgent need for targeted mental health and organizational support interventions to improve nurse retention and well-being, particularly in resource-constrained healthcare settings. Future research should consider broader socio-political factors influencing nurses’ work environments in Lebanon.

## Introduction

The Novel Coronavirus 2019, also known as COVID-19, has been circulating in the news since early 2020 (1). Even though the coronavirus has been existing for a while, COVID-19 is a novel variant that has created widespread alarm (2, 3), harm to global health, and losses in the macroeconomic industries worldwide (4). This new coronavirus variant is more dangerous for those with weakened immune systems. The severe acute respiratory syndrome (SARS) Coronavirus 2 (SARS-CoV-2) infection was brought on by the COVID-19 outbreak (5), with millions of people infected with COVID-19 till now (6). Consequently, on January 30, 2020, the World Health Organization declared it a global health emergency (7). Due to the magnitude of the situation, massive numbers of medical personnel were sent to the affected areas in order to save, treat, and contain the COVID-19 outbreak.

Occupational stress brought on by the potential COVID-19 infection has been largely covered in phenomenological analysis reflecting on nurses’ past experiences rather than being investigated in empirical studies. According to previous organizational psychology research, workplace stress is a sign of a growing amount of work, a deteriorating mental status, and a worsening job conditions. Studies have revealed a strong correlation between job stress and symptoms of anxiety, psychosis, and emotional exhaustion (8–10). Due to a similar correlation indicating that higher levels of occupational stress associated with higher levels of emotional fatigue, which may increase nurses intention to leave their jobs, the COVID-19 outbreak has increased the amount of job strain and ambiguity among nursing staff (11, 12). Caring for COVID-19 patients puts nurses at greater risk for contamination and perhaps death. According to previous SARS investigations, nurses who were placed under confinement experienced higher levels of post-traumatic stress disorder, nervousness, personal and social exclusion, fear of spreading the disease to others, and social alienation. They also experienced higher levels of dissatisfaction, anger, lack of control, and apparent stigma (13, 14). Given the paucity of studies on COVID-19-related occupational stress and frustration, nurses themselves are inexperienced with dealing with the psychological trauma of the worldwide COVID-19 outbreak. Additionally, because of the prevalent COVID-19 outbreak, numerous nurse practitioners are feeling stressed out and dissatisfied at work (15, 16), and other nurses are unwilling to even provide care services. Parallel to this, factors like workplace stress and dissatisfaction are associated with emotional fatigue among nursing professionals, which may contribute to increased intent to quit (17, 18).

Nursing staff play a main role in the healthcare teams, specially in comprehending the urgency and scope of care delivery required to handle patients most effectively during the propagation of COVID-19 globally (19). Consequently, the institution and executives who want to maintain a skilled and competent nursing staff are greatly impacted by nurse turnover (20). During epidemics, nursing attrition is a major problem for the delivery of healthcare.

This study contributes to the existing knowledge by evaluating the connection between occupational stress, job satisfaction, and intent to leave throughout the COVID-19 pandemic. The findings may aid in comprehending the phenomenon of workplace stress during the COVID-19 epidemic (21). This research cleared the way for a deeper comprehension of the processes at play when negative circumstances influence an employee’s intentions to leave their job (22). Additionally, according to Guixia and Hui (23), burnout and job stress cause a variety of withdrawal reactions that have a negatively impact the health sector. Additionally, the study contributes empirical data demonstrating that increased job stress and decreased job satisfaction due to COVID-19 were associated with higher levels of burnout, turnover intention, and psychological distress. Critical care units at hospitals can leverage a better comprehension of the interactions between various elements impacting turnover intention to create policies and procedures aimed at boosting nurse job stability. Nursing supervisors need to be aware of the occupational stress, satisfaction, and emotional exhaustion that nurses are experiencing in relation to the widespread COVID-19 outbreak (24, 25). Although research on nurses’ desire to leave the profession are few, those that exist tend to concentrate on this intention in everyday circumstances (26). No research has been done on nurses’ intentions to leave the profession as a result of the stress and work dissatisfaction they encountered during COVID-19 in Lebanon. This study was inspired by the need for more research in this specific field, and it is characteristically important in the Lebanese context especially in the past 3 years where the population has been facing concurrent political, economic and health crises which have been endangering the livelihood of the citizens. Healthcare providers and mainly nurses have been significantly impacted by the crises as their practice has been restricted by the lack of medical supplies and medications due to currency shortage, lack of subsidization, and challenging importing procedures. In addition, the salaries the nurses have been acquiring recently is not enough to maintain an adequate lifestyle which is also demotivating them to remain practicing nursing in Lebanon, and notably large numbers of nurses and doctors have been leaving since 2019, which makes this study of extreme importance.

### Aim

The aim of the present study was to examine the relationship between occupational stress, job satisfaction, and intent to leave among nurses dealing with COVID-19 patients in Lebanon.

### Research question

Is there a relationship between occupational stress, job satisfaction, and intent to leave among nurses dealing with COVID-19 patients in Lebanon?

## Materials and methods

### Design

This study was conducted using a cross-sectional correlational descriptive research design.

### Setting

This research was carried out amongst critical care nurses practicing in the Cardiac Care Unit (CCU), Intensive Care Unit (ICU), and Emergency Room (ER) at a single university hospital in Lebanon. This hospital was selected as the study setting because it is a university-affiliated hospital that provides a suitable environment for clinical research and practice.

### Population and sampling

The sample of this study included registered bedside nurses working in the critical care areas including Intensive care unit (ICU), Cardiac intensive care unit (CICU), Pediatric intensive care unit (PICU) and Neonatal intensive care unit (NICU), emergency room (ER). Critical care professionals who were already employed in the approached clinical setting, completed the eligibility requirements, and agreed freely to participate in the research were enrolled in the convenience sample used in the investigation. Using a convenience sample requires the investigator to take into account every potential respondent they come across throughout the recruiting process. The investigator had to enroll all of the available and accessible respondents since there were so few critical care nurses who were accessible for the research which makes the convenience sample strategy justified. The sample size determined using G-power version 3.1. A medium-effect size for ANOVA 0.25 and a significance level of 0.05, power of 0.8, which is commonly used in nursing research, resulting in an estimated sample size of 140. The researcher enrolled all available and accessible respondents from a population of 140 registered critical care working at the selected university hospital. The final sample consisted of 112 nurses, exceeding the necessary effect size.

#### Inclusion criteria

The study included registered bedside nurses who worked full-time and had at least 6 months of experience in critical care units. This minimum experience requirement was recognized to ensure that nurses were appropriately prepared and familiar with operational conditions in critical care areas.

#### Exclusion criteria

Nurses were excluded from the study if they were on any type of leave during the data collection period (e.g., sick leave, maternity leave), working in administrative or non-clinical roles, or not directly involved in patient care. In addition, nurses who had a confirmed COVID-19 infection during the data collection period were excluded to minimize potential bias related to personal health status.

### Tools

Data collection was conducted using an electronic survey including the following sections:

*Part I* (Introduction): Brief overview of the context, aim, voluntary participation, procedures, anonymity and confidentiality declarations, and selection criteria.

*Part II* (Sociodemographic characteristics and employment background): Age, gender, marital status, level of education, years of experience, average job hours, and the number of nights shifts.

*Part III* (COVID-19 stressors): were obtained and adapted from the “MERS-CoV staff survey” by Khalid et al. (27) and the US National Center for Posttraumatic Stress Disorder 2020. It has 23 questions on a 5-point Likert scale with 0 being never exposed to stress, 1 meaning never stressful, 2 meaning occasionally stressful, 3 meaning regularly stressful, and 4 meaning highly stressful. The elements are broken down into four subscales: stigma (2 items min. Score = 0, max. Score = 8), exposure to infection risk (5 items min. Score = 0, max. Score = 20), personal demands and fears (6 items min. Score = 0, max. Score = 24), and the need to employ strict biosecurity measures (10 items min. Score = 0, max. Score = 40). Greater scores suggest elevated stress degrees. For every subscale, the person’s answers to each question are assessed and added up to provide a total score. The total score of all subscales was added to determine the amount of particular COVID-19-associated stress, which was then divided into three categories: low (total score 23), moderate (24–48), and high (49–92). Cronbach’s Alpha was used in a reliability test, and the result was 0.89.

The MERS-CoV Staff Survey was culturally adapted to ensure its suitability for the Lebanese healthcare context. The adaptation process involved expert review by two nursing academics and one public health specialist to assess content relevance and clarity. Minor modifications were made to wording to reflect the COVID-19 context and local clinical practices, while preserving the original meaning of the items. No major structural changes were made to the instrument, and the core domains of the original scale were retained to maintain comparability with previous studies.

*Part IV* (Job Satisfaction): Measured using the McCloskey/Mueller Satisfaction Scale (MMSS), a widely used, valid, and reliable instrument for assessing job satisfaction among nursing staff. It has 25 items on a 5-point Likert scale, with 1 being very discontent, 2 denoting moderate dissatisfaction, 3 denoting neither satisfaction nor dissatisfaction, 4 denoting moderate satisfaction, and 5 denoting high satisfaction (50). The items are grouped into eight subscales: (1) extrinsic rewards (3 items, min. Score = 1, max. Score = 15), (2) family and work balance (3 items, min. Score = 1, max. Score = 15), (3) professional opportunities (4 items, min. Score = 4, max. Score = 20), (4) work control and responsibility (4 items, min. Score = 4, max. Score = 20), (5) scheduling (6 items, min. Score = 1, max. Score = 30), (6) coworkers (2 items, min. Score = 1, max. Score = 10), (7) interaction opportunities (2 items, min. Score = 2, max. Score = 10), and (8) praise and recognition (4 items, min. Score = 1, max. Score = 20) (28). Higher scores indicated greater satisfaction rates when calculating the mean score for each subscale and the full survey. Additionally, the total score of all sub dimensions was added to determine the level of job satisfaction, which was then divided into three categories: low (total score 52), moderate (53–104), and high (105–125). The Cronbach alpha reliability coefficient was 0.82.

*Part V* (The nurses’ intention to leave): Assessed using the question “If feasible, would you quit your present institution within the next year as a consequence of job dissatisfaction?” with dichotomous responses (yes/no). If responded yes, they were asked a follow-up question about what do you intend to leave? was the next query (only included in the descriptive analysis), with the alternatives “the present position” “the current organization” and “the field of nursing” as possible answers (29).

All study instruments were administered in English, which is the primary language of clinical education and professional communication among nurses in Lebanon. The use of English-based instruments is common in similar studies conducted in the region.

The reliability of the instruments was assessed in the current study using Cronbach’s alpha coefficients. The MERS-CoV stress scale demonstrated good internal consistency (*α* = 0.89), and the McCloskey/Mueller Satisfaction Scale showed acceptable reliability (α = 0.82).

### Method

1. An IRB approval was obtained from the responsible authorities at the hospital where the study was conducted.
2. An informed consent was obtained from nurses after explanation of the aim of the study. The informed consent form included the details of the research study such as aim of the study, benefits of participation, risks which there were not any considering it was a descriptive study, opt out and confidentiality policy as well as contact information of the researcher.
3. A total of 140 eligible nurses working in the selected hospital were invited to participate in the study. Of these, 112 nurses completed the questionnaire, resulting in a response rate of approximately 80%. Participation was entirely voluntary, and no incentives were provided. The survey was distributed electronically using Google Forms. All responses were collected anonymously, and no identifying information (such as names, employee numbers, or contact details) was obtained. This ensured confidentiality and minimized the risk of participant identification. Although the study was conducted in a single hospital, the selected setting represents a major healthcare institution providing critical care services, which enhances the relevance of the findings.

### Ethical considerations

Ethical approval for this study was obtained from the Ethics Committee of Haykel Hospital (Approval No. ECO-R-815, dated [2/6/2022]). Participation in the study was voluntary, and written informed consent was obtained from all participants prior to data collection. Participants were informed of their right to withdraw from the study at any time without any consequences. Although the study involved minimal psychological risk, given that it addressed topics related to occupational stress and job satisfaction, participants were informed about the potential for mild emotional discomfort. Information regarding available psychological support services within the hospital was provided in case participants experienced any distress. All data were collected anonymously using an online questionnaire. No identifying information (such as names, employee numbers, or contact details) was collected. Given that the study was conducted within a single hospital, additional care was taken to ensure that individual responses could not be traced back to specific participants.

Data were stored securely on password-protected devices accessible only to the research team. The data will be retained for a limited period in accordance with institutional policies and then permanently deleted to ensure confidentiality.

### Statistical analysis

This research used a quantitative cross-sectional correlational design to address the research questions. Inferential statistics and descriptive analysis were both used, along with independent *T*-tests, ANOVA tests, and Pearson’s correlation. Illustrative tables were created using SPSS version 25 for data entry and analysis.

## Results

### Participant characteristics

The sample of this study comprised 112 critical care nurses, where 45 (40.2%) males, and 67 (59.8%) females. The descriptive statistics showed that the majority of the sample held a bachelor degree in nursing (58%), while 14 (12.5%) held a technical diploma, and 31 (27.7%) held a master degree in nursing. In addition, 40 (35.7%) of the nurses were single, while 70 (62.5%) were married, and only 2 (1.8%) were divorced. The age distribution showed that 32 (28.6%) were between 26 and 30 years old, 26 (23.2%) were between 36 and 45 years old, and 25 (22.3%) were between18 and 25 years of age. The majority of the nurses 46 (41.1%) had 6 to 10 years of experience. The analysis also showed that the mean working hours were 44.54 ± 13.90 h per week, with an average of 1.87 ± 3.71-night duties per week (Table 1).

**Table 1:** Participant characteristics.

Variables	Category	*N*	%
Gender	Male	45	40.2
	Female	67	59.8
Educational degree	TS	14	12.5
	BT	2	1.8
	BS	65	58.0
	MS	31	27.7
Marital Status	Single	40	35.7
	Married	70	62.5
	Divorced	2	1.8
Age	18–25 years	25	22.3
	26–30 years	32	28.6
	31–35 years	23	20.5
	36–45 years	26	23.2
Years of experience	6 months to 1 year	31	27.7
	1 to 3 years	18	16.1
	3 to 6 years	17	15.2
	6 to 10 years	46	41.1
	Mean	SD	
Work Hours per week	44.54	13.90	
Night duties per week	1.87	3.71	

### Descriptive statistics of the main variables

#### COVID-19 stressors

Descriptive analysis was carried out to determine the level of occupational stress relating to COVID-19 that was prevalent among the participating critical care nurses. The results showed that the nurses scored an above average level mean of 4.26 ± 1.93 on the level of stigma of COVID-19 infection, 13.85 ± 3.62 on the level of stress relating to the risk of infection, 14.23 ± 4.99 on the level of stress relating to personal demands and fears, and 25.62 ± 7.83 on the level of stress relating to the application of biosecurity measures. The nurses scored a mean of 57.96 ± 15.10 on the level of the total stress score which is between 49 and 92, thus qualifying as a high level of stress (Table 2).

**Table 2:** COVID-19 stressors subscales.

	*N*	Min	Max	Mean	SD
Stigma	112	0.00	8.00	4.26	1.93
Risk of infection	112	0.00	20.00	13.85	3.62
Personal demands and fears	112	0.00	24.00	14.23	4.99
Biosecurity	112	0.00	40.00	25.62	7.83
Total Stress	112	23.00	92.00	57.96	15.10

#### Job satisfaction

Participants reported varying levels of satisfaction across different domains The results show that the nurses scored a rather high mean of 13.38 ± 3.40 on the level of praise and recognition, a moderate mean of 19.70 ± 5.41 on the level of scheduling, a similar mean of 6.23 ± 1.82 on the level of interaction opportunities, 11.75 ± 2.94 on the level of professional opportunities, 13.34 ± 3.12 on the level of work control, 9.35 ± 2.70 on the level of family-work balance, 8.00 ± 2.87 on the level of extrinsic rewards, and 7.04 ± 1.58 on the level of relationship with coworkers. The nurses notably scored a mean of 79.37 ± 16.10 on the level of the total satisfaction score which is between 53 and 104 which is considered a moderate level of satisfaction (Table 3).

**Table 3:** Satisfaction subscales.

	*N*	Min	Max	Mean	SD
Praise and recognition	112	1.00	15.00	13.38	3.40
Scheduling	112	1.00	30.00	19.70	5.41
Interaction opportunities	112	1.00	10.00	6.23	1.82
Professional opportunities	112	1.00	20.00	11.75	2.94
Work control	112	1.00	20.00	13.34	3.12
Family and work balance	112	1.00	15.00	9.35	2.70
Extrinsic rewards	112	1.00	15.00	8.00	2.87
Coworkers	112	1.00	10.00	7.04	1.58
Total satisfaction score	112	1.00	125.00	79.37	16.10

#### Intent to leave

The nurses were asked if they intend to leave their current job, and 63 (56.3%) of them indicated that they would, where 35 (31.3%) would leave the organization, 22 (19.6%) would leave their current position, and 19 (17%) would leave the nursing profession as a whole (Table 4).

**Table 4:** Intent to leave.

Variable	Category	*N*	%
Would you leave your current job?	No	49	43.8
	Yes	63	56.3
If yes, what do you intend to leave?	None	36	32.1
	The current position	22	19.6
	The current organization	35	31.3
	The field of nursing	19	17.0

#### Differences in study variables by demographic characteristics

Independent *t*-tests revealed no significant differences between males and females regarding stress and satisfaction scores across all subscales and total scores (Table 5).

**Table 5:** Difference in study variables according to gender.

	Gender	*N*	Mean	SD	*p*-value
Stigma	Male	45	3.71	1.85	0.91
	Female	67	4.63	1.91	
Risk of infection	Male	45	13.22	3.72	0.88
	Female	67	14.27	3.51	
Personal demands and fears	Male	45	14.00	4.82	0.38
	Female	67	14.39	5.13	
Biosecurity	Male	45	24.20	7.83	0.96
	Female	67	26.57	7.74	
Total stress score	Male	45	55.13	14.96	0.67
	Female	67	59.85	15.00	
Praise and recognition	Male	45	13.18	3.52	0.44
	Female	67	13.51	3.33	
Scheduling	Male	45	20.22	4.66	0.06
	Female	67	19.34	5.87	
Interaction opportunities	Male	45	6.13	1.88	0.77
	Female	67	6.30	1.79	
Professional opportunities	Male	45	11.73	2.74	0.34
	Female	67	11.76	3.09	
Work control	Male	45	13.27	3.09	0.95
	Female	67	13.39	3.17	
Family-work balance	Male	45	9.38	2.77	0.66
	Female	67	9.33	2.67	
Extrinsic rewards	Male	45	8.18	3.03	0.40
	Female	67	7.88	2.78	
Coworkers	Male	45	7.22	1.52	0.58
	Female	67	6.91	1.62	
Total satisfaction	Male	45	79.76	15.27	0.60
	Female	67	79.10	16.74	

ANOVA tests examining differences by age showed a significant difference in stress related to infection risk (*p* = 0.01), with nurses aged 31–35 years reporting the highest levels (15.04 ± 4.04). Similarly, satisfaction with scheduling differed significantly by age (*p* = 0.03), with nurses aged 36–45 years reporting the highest satisfaction (21.76 ± 3.74) (Table 6).

**Table 6:** Difference in stress scores according to age.

		*N*	Mean	SD	*p*-value
Stigma	18–25 years	25	4.28	2.07	0.80
	26–30 years	32	3.97	1.66	
	31–35 years	23	4.52	2.23	
	36–45 years	26	4.23	2.07	
	> 45 years	6	4.83	0.75	
	Total	112	4.26	1.93	
Risk of infection	18–25 years	25	14.80	2.84	0.01*
	26–30 years	32	12.91	4.28	
	31–35 years	23	15.04	3.04	
	36–45 years	26	13.85	3.31	
	> 45 years	6	10.33	3.33	
	Total	112	13.85	3.62	
Personal demands and fears	18–25 years	25	14.72	4.62	0.90
	26–30 years	32	13.84	4.54	
	31–35 years	23	14.70	5.50	
	36–45 years	26	14.15	5.61	
	> 45 years	6	12.83	5.15	
	Total	112	14.23	4.99	
Biosecurity	18–25 years	25	25.84	6.88	0.76
	26–30 years	32	25.91	7.52	
	31–35 years	23	25.22	9.55	
	36–45 years	26	26.31	7.80	
	> 45 years	6	21.67	7.37	
	Total	112	25.62	7.83	
Total stress score	18–25 years	25	59.64	13.49	0.62
	26–30 years	32	56.63	13.83	
	31–35 years	23	59.48	18.30	
	36–45 years	26	58.54	15.95	
	> 45 years	6	49.67	11.67	
	Total	112	57.96	15.10	

Bold values indicate statistically significant findings, and “*” indicates *p* <0.05.

An ANOVA test was carried out to determine if there is a difference in satisfaction means according to the different ages of the nurses. A significant difference was prevalent on the level of satisfaction with scheduling (*p* = 0.03), where nurses between 36 and 45 years of age scored the highest mean (21.76 ± 3.74) (Table 7).

**Table 7:** Difference in satisfaction scores according to age.

Praise and recognition	18–25 years	25	13.40	3.39	0.32
	26–30 years	32	12.41	3.48	
	31–35 years	23	14.13	2.87	
	36–45 years	26	13.58	3.28	
	> 45 years	6	14.67	5.05	
	Total	112	13.38	3.40	
Scheduling	18–25 years	25	17.80	5.47	0.03*
	26–30 years	32	18.41	6.43	
	31–35 years	23	21.17	4.47	
	36–45 years	26	21.77	3.74	
	> 45 years	6	19.83	5.64	
	Total	112	19.70	5.41	
Interaction opportunities	18–25 years	25	6.72	1.72	0.62
	26–30 years	32	6.00	1.85	
	31–35 years	23	6.17	1.95	
	36–45 years	26	6.19	1.65	
	> 45 years	6	5.83	2.48	
	Total	112	6.23	1.82	
Professional opportunities	18–25 years	25	11.60	3.19	0.52
	26–30 years	32	11.34	3.39	
	31–35 years	23	12.65	1.99	
	36–45 years	26	11.77	2.72	
	> 45 years	6	11.00	3.41	
	Total	112	11.75	2.94	
Work control	18–25 years	25	12.96	3.37	0.63
	26–30 years	32	12.88	3.34	
	31–35 years	23	14.00	2.59	
	36–45 years	26	13.50	3.15	
	> 45 years	6	14.17	2.93	
	Total	112	13.34	3.12	
Family work balance	18–25 years	25	8.64	2.66	0.47
	26–30 years	32	9.19	3.27	
	31–35 years	23	9.48	2.09	
	36–45 years	26	9.96	2.32	
	> 45 years	6	10.00	3.16	
	Total	112	9.35	2.70	
Extrinsic rewards	18–25 years	25	7.24	2.98	0.62
	26–30 years	32	8.25	3.30	
	31–35 years	23	8.30	2.55	
	36–45 years	26	8.27	2.46	
	> 45 years	6	7.50	3.15	
	Total	112	8.00	2.87	
Coworkers	18–25 years	25	6.96	1.86	0.52
	26–30 years	32	6.75	1.63	
	31–35 years	23	7.39	1.31	
	36–45 years	26	7.00	1.30	
	> 45 years	6	7.67	2.25	
	Total	112	7.04	1.58	
Total satisfaction	18–25 years	25	76.72	16.56	0.36
	26–30 years	32	76.00	19.42	
	31–35 years	23	83.57	11.08	
	36–45 years	26	82.04	13.69	
	> 45 years	6	80.67	19.85	
	Total	112	79.37	16.10	

Bold values indicate statistically significant findings, and “*” indicates *p* <0.05.

Regarding years of experience, no significant differences were found in stress scores. However, satisfaction with scheduling differed significantly by experience (*p* < 0.001), with nurses having 6–10 years of experience reporting the highest satisfaction (21.73 ± 4.29) (Tables 8, 9).

**Table 8:** Difference in stress scores according to experience.

		*N*	Mean	SD	*p*-value
Stigma	6 months to 1 year	31	4.03	2.09	0.23
	1 to 3 years	18	5.11	2.05	
	3 to 6 years	17	4.12	1.65	
	6 to 10 years	46	4.13	1.83	
	Total	112	4.26	1.93	
Risk of infection	6 months to 1 year	31	14.03	3.89	0.89
	1 to 3 years	18	14.22	4.39	
	3 to 6 years	17	13.35	3.76	
	6 to 10 years	46	13.76	3.11	
	Total	112	13.85	3.62	
Personal demands and fears	6 months to 1 year	31	14.10	5.15	0.26
	1 to 3 years	18	15.94	4.22	
	3 to 6 years	17	15.06	4.53	
	6 to 10 years	46	13.35	5.25	
	Total	112	14.23	4.99	
Biosecurity	6 months to 1 year	31	25.97	7.39	0.21
	1 to 3 years	18	28.44	8.37	
	3 to 6 years	17	26.35	7.61	
	6 to 10 years	46	24.00	7.85	
	Total	112	25.62	7.83	
Total stress score	6 months to 1 year	31	58.13	14.69	0.24
	1 to 3 years	18	63.72	17.11	
	3 to 6 years	17	58.88	13.12	
	6 to 10 years	46	55.24	15.01	
	Total	112	57.96	15.10	

**Table 9:** Difference in satisfaction scores according to experience.

		*N*	Mean	SD	*p*-value
Praise and recognition	6 months to 1 year	31	13.0000	3.42540	0.16
	1 to 3 years	18	14.3333	3.59738	
	3 to 6 years	17	12.0000	3.37268	
	6 to 10 years	46	13.7609	3.22618	
	Total	112	13.3750	3.39879	
Scheduling	6 months to 1 year	31	18.5806	5.90353	0.00*
	1 to 3 years	18	18.8333	5.76245	
	3 to 6 years	17	17.1176	5.32544	
	6 to 10 years	46	21.7391	4.29695	
	Total	112	19.6964	5.41243	
Interaction opportunities	6 months to 1 year	31	6.6129	1.97783	0.12
	1 to 3 years	18	6.8333	1.82305	
	3 to 6 years	17	5.8235	1.70423	
	6 to 10 years	46	5.8913	1.68955	
	Total	112	6.2321	1.82071	
Professional opportunities	6 months to 1 year	31	11.1935	3.30070	0.28
	1 to 3 years	18	12.7778	3.02063	
	3 to 6 years	17	11.2941	3.13777	
	6 to 10 years	46	11.8913	2.52303	
	Total	112	11.7500	2.93933	
Work control	6 months to 1 year	31	12.6452	2.95012	0.12
	1 to 3 years	18	14.7222	3.73860	
	3 to 6 years	17	12.7647	3.26974	
	6 to 10 years	46	13.4783	2.81850	
	Total	112	13.3393	3.12374	
Family work balance	6 months to 1 year	31	9.2258	2.99677	0.43
	1 to 3 years	18	9.4444	3.05291	
	3 to 6 years	17	8.4706	2.69531	
	6 to 10 years	46	9.7174	2.32535	
	Total	112	9.3482	2.69704	
Extrinsic rewards	6 months to 1 year	31	8.0645	3.41502	0.63
	1 to 3 years	18	8.3333	3.02927	
	3 to 6 years	17	7.1765	2.65130	
	6 to 10 years	46	8.1304	2.50873	
	Total	112	8.0000	2.87267	
Coworkers	6 months to 1 year	31	6.9032	1.70009	0.58
	1 to 3 years	18	7.3889	1.75361	
	3 to 6 years	17	6.7059	1.49016	
	6 to 10 years	46	7.1087	1.47916	
	Total	112	7.0357	1.58216	
Total satisfaction	6 months to 1 year	31	77.2581	17.08990	0.15
	1 to 3 years	18	82.8889	17.42510	
	3 to 6 years	17	72.8235	17.78073	
	6 to 10 years	46	81.8261	13.67448	
	Total	112	79.3661	16.09767	

Bold values indicate statistically significant findings, and “*” indicates *p* <0.05.

Analysis by marital status revealed no significant differences in stress levels. However, satisfaction with scheduling differed significantly (*p* = 0.01), with married nurses reporting the highest satisfaction (20.83 ± 5.24). Additionally, satisfaction with coworker relationships differed significantly (*p* < 0.05), with single nurses reporting the highest satisfaction (7.17 ± 1.50) (Tables 10, 11).

**Table 10:** Difference in stress scores according to marital status.

		*N*	Mean	SD	*p*-value
Stigma	Single	40	4.08	1.93	0.16
	Married	70	4.43	1.89	
	Divorced	2	2.00	2.83	
	Total	112	4.26	1.93	
Risk of infection	Single	40	13.70	3.94	0.78
	Married	70	13.89	3.40	
	Divorced	2	15.50	6.36	
	Total	112	13.85	3.62	
Personal demands and fears	Single	40	14.30	4.76	0.46
	Married	70	14.07	5.16	
	Divorced	2	18.50	2.12	
	Total	112	14.23	4.99	
Biosecurity	Single	40	24.48	8.49	0.16
	Married	70	26.01	7.33	
	Divorced	2	34.50	7.78	
	Total	112	25.62	7.83	
Total stress score	Single	40	56.55	16.13	0.41
	Married	70	58.40	14.53	
	Divorced	2	70.50	13.44	
	Total	112	57.96	15.10	

**Table 11:** Difference in satisfaction scores according to marital status.

		*N*	Mean	SD	*p*-value
Praise and recognition	Single	40	12.88	3.41	0.27
	Married	70	13.73	3.36	
	Divorced	2	11.00	4.24	
	Total	112	13.38	3.40	
Scheduling	Single	40	17.93	4.96	0.01*
	Married	70	20.83	5.24	
	Divorced	2	15.50	12.02	
	Total	112	19.70	5.41	
Interaction opportunities	Single	40	6.55	1.68	0.18
	Married	70	6.10	1.87	
	Divorced	2	4.50	2.12	
	Total	112	6.23	1.82	
Professional opportunities	Single	40	11.68	2.93	0.39
	Married	70	11.87	2.92	
	Divorced	2	9.00	4.24	
	Total	112	11.75	2.94	
Work control	Single	40	12.85	3.07	0.07
	Married	70	13.73	3.08	
	Divorced	2	9.50	3.54	
	Total	112	13.34	3.12	
Family work balance	Single	70	9.73	2.81	0.15
	Married	2	8.50	3.54	
	Divorced	112	9.35	2.70	
	Total	40	7.45	2.84	
Extrinsic rewards	Single	70	8.37	2.86	0.16
	Married	2	6.00	2.83	
	Divorced	112	8.00	2.87	
	Total	40	6.93	1.62	
Coworkers	Single	70	7.17	1.50	**0.05**
	Married	2	4.50	2.12	
	Divorced	112	7.04	1.58	
	Total	40	76.38	15.97	
Total satisfaction	Single	70	81.61	15.45	0.06
	Married	2	60.50	30.41	
	Divorced	112	79.37	16.10	
	Total	40	12.88	3.41	

Bold values indicate statistically significant findings, and “*” indicates *p* <0.05.

#### Bivariate analysis

A Pearson’s correlation test was carried out to determine if there is a relationship between the study variables and the characteristics of the participating nurses. The results showed a significant correlation between gender and stress of stigma (*p* = 0.01). Satisfaction with scheduling was correlated with years of experience (*p* = 0.01) marital status (*p* = 0.03), and educational level (*p* = 0.01) while satisfaction with family-work balance was correlated with the number of working hours per week (*p* = 0.02), where a negative relationship was prevalent, thus the more hours were there the lower the satisfaction might be. A significant correlation was also found between age and extrinsic rewards (*p* = 0.02) (Table 12).

**Table 12:** Correlation between study variables and nurse characteristics.

		Gender	Work hours per week	Night duties per week	Years of experience	Marital status	Age	Education level
Stigma	R-value	0.23	−0.04	−0.03	−0.03	0.03	−0.01	0.06
	*p*-value	0.01	0.70	0.78	0.78	0.79	0.95	0.56
Risk of infection	R-value	0.14	0.13	0.04	−0.04	0.05	0.15	−0.12
	*p*-value	0.13	0.16	0.65	0.65	0.64	0.12	0.20
Personal demands and fears	R-value	0.04	0.09	0.13	−0.09	0.02	0.01	−0.05
	*p*-value	0.69	0.34	0.18	0.35	0.83	0.95	0.63
Biosecurity	R-value	0.15	−0.05	0.13	−0.13	0.14	0.01	−0.05
	*p*-value	0.12	0.63	0.16	0.16	0.13	0.90	0.62
Total Stress	R-value	0.15	0.03	0.12	−0.11	0.10	0.04	−0.06
	*p*-value	0.11	0.73	0.21	0.24	0.32	0.66	0.52
Praise and recognition	R-value	0.05	−0.10	−0.05	0.05	0.08	−0.05	0.11
	*p*-value	0.62	0.31	0.60	0.60	0.41	0.57	0.25
Scheduling	R-value	−0.08	−0.20	−0.20	0.24	0.20	−0.05	0.26
	*p*-value	0.40	0.04	0.03	0.01	0.03	0.60	0.01
Interaction opportunities	R-value	0.05	−0.01	0.08	−0.20	−0.16	−0.19	−0.09
	*p*-value	0.64	0.94	0.39	0.04	0.10	0.05	0.33
Professional opportunities	R-value	0.01	0.09	0.06	0.06	−0.02	−0.21	0.03
	*p*-value	0.96	0.33	0.57	0.57	0.88	0.03	0.75
Work control	R-value	0.02	−0.10	−0.02	0.06	0.07	−0.08	0.11
	*p*-value	0.84	0.30	0.83	0.56	0.48	0.39	0.23
Family-work balance	R-value	−0.01	−0.22	−0.04	0.06	0.15	−0.11	0.18
	*p*-value	0.93	0.02	0.69	0.53	0.11	0.24	0.06
Extrinsic rewards	R-value	−0.05	−0.07	0.09	−0.01	0.11	−0.22	0.08
	*p*-value	0.59	0.44	0.35	0.92	0.25	0.02	0.40
Coworkers	R-value	−0.10	−0.10	0.05	0.02	−0.01	−0.03	0.09
	*p*-value	0.31	0.29	0.62	0.81	0.94	0.79	0.34
Total satisfaction	R-value	−0.02	−0.12	−0.04	0.08	0.09	−0.14	0.15
	*p*-value	0.84	0.20	0.72	0.40	0.35	0.14	0.12

Another bivariate correlational analysis was conducted to examine the relationship between study variables. The results showed that there was a significant negative correlation between stress relating to stigma of infection and interaction opportunities (*p* = 0.00), professional opportunities (*p* = 0.00), work control (*p* = 0.00), relationship with co-workers (P0.02), and the total satisfaction score (*p* = 0.00) (Table 13).

**Table 13:** Correlation between study variables.

		Stigma	Risk of infection	Personal demands and fears	Biosecurity	Total stress
Scheduling	R-value	0.05	−0.03	−0.09	−0.07	−0.07
	*p*-value	0.61	0.77	0.32	0.47	0.48
Interaction opportunities	R-value	−0.28	0.06	0.18	0.12	0.17
	*p*-value	**0.00**	0.50	0.06	0.20	0.07
Professional opportunities	R-value	−0.28	0.03	0.07	0.02	0.07
	*p*-value	**0.00**	0.79	0.49	0.87	0.45
Work control	R-value	−0.31	−0.09	0.08	0.03	0.06
	*p*-value	**0.00**	0.36	0.42	0.72	0.52
Family work balance	R-value	0.10	−0.09	−0.04	0.02	−0.01
	*p*-value	0.31	0.34	0.65	0.82	0.90
Extrinsic rewards	R-value	0.18	−0.09	0.07	0.16	0.11
	*p*-value	0.06	0.35	0.45	0.09	0.25
Coworkers	R-value	−0.22	0.02	0.05	0.04	0.07
	*p*-value	**0.02**	0.85	0.63	0.69	0.48
Total satisfaction	R-value	−0.27	−0.03	0.06	0.05	0.08
	*p*-value	**0.00**	0.79	0.54	0.58	0.43

Bold values indicate statistically significant findings.

#### Regression analysis

A regression analysis was conducted to determine the predictors of occupational stress relating to COVID-19. The results showed that marital status predicted the scored levels of stress (*p* = 0.04) (Table 14).

**Table 14:** Predictors of stress.

Model		Unstandardized coefficients		Standardized coefficients	*t*	Sig.
		B	Std. error	Beta		
1	(Constant)	47.54	7.32		6.50	0.00
	Gender	4.83	3.04	0.16	1.59	0.12
	Age	−0.84	1.82	−0.07	−0.46	0.65
	Years of experience	−1.90	1.71	−0.16	−1.11	0.27
	Marital Status	7.44	3.47	0.25	2.14	0.04*
	Education Level	1.90	1.66	0.11	1.14	0.26
	Work Hours per week	0.06	0.11	0.05	0.52	0.60
	Night duties per week	0.46	0.41	0.11	1.13	0.26

Bold values indicate statistically significant findings, and “*” indicates *p* <0.05.

The results also showed that education level predicted higher satisfaction (*p* = 0.05) (Table 15).

**Table 15:** Predictors of satisfaction.

Model		Unstandardized coefficients		Standardized coefficients	*t*	Sig.
		B	Std. error	Beta		
1	(Constant)	90.63	7.88		11.51	0.00
	Gender	−2.27	3.27	−0.07	−0.70	0.49
	Age	3.15	1.96	0.24	1.61	0.11
	Years of experience	−0.81	1.85	−0.06	−0.44	0.66
	Marital status	−0.50	3.74	−0.02	−0.13	0.90
	Education level	−3.53	1.79	−0.20	−1.97	**0.05**
	Work hours per week	−0.14	0.12	−0.12	−1.24	0.22
	Night duties per week	0.10	0.44	0.02	0.22	0.83

Bold values indicate statistically significant findings.

As for intent to leave, the results showed that age was a significant predictor (*p* = 0.05), and satisfaction scores also predicted the intent to leave (*p* = 0.00) (Table 16).

**Table 16:** Predictors of intent to leave.

Model		Unstandardized coefficients		Standardized coefficients	*t*	Sig.
		B	Std. error	Beta		
1	(Constant)	0.46	0.24		1.88	0.06
	Gender	0.03	0.10	0.03	0.29	0.77
	Age	−0.12	0.06	−0.30	−2.00	0.05
	Years of experience	0.02	0.06	0.04	0.26	0.80
	Marital status	0.15	0.12	0.16	1.31	0.19
	Education level	0.07	0.06	0.13	1.28	0.20
	Work hours per week	0.00	0.00	−0.01	−0.10	0.92
	Night duties per week	0.02	0.01	0.14	1.35	0.18
	Stress	0.00	0.00	0.12	1.32	0.19
	Satisfaction	−0.01	0.00	−0.29	−3.14	**0.00**

Bold values indicate statistically significant findings.

## Discussion

The COVID-19 outbreak may place very severe mental strain on those practicing in hospitals. The goal of the present research was to outline the professional traits of critical care unit nurses after they had worked for around 2 years during the SARS-CoV-2 outbreak. It was predicted that the participants would have greater degrees of occupational stress and job dissatisfaction indicators predicated upon encounters working during the epidemic and the research that was accessible (30).

The findings of this study indicate that critical care nurses experienced high levels of occupational stress during the COVID-19 pandemic. These findings are consistent with previous international studies conducted in Egypt and China, which reported elevated stress levels among frontline nurses during the pandemic (31, 32). Similarly, a systematic review by Galanis et al. (33) highlighted that healthcare workers, particularly nurses, experienced high levels of stress, anxiety, and burnout during the pandemic (34). These findings are further supported by recent evidence highlighting occupational stressors and coping challenges among critical care nurses (35).

However, the stress levels observed in the current study may be comparatively higher, which could be attributed to the compounded socio-economic and political crises in Lebanon. Unlike more stable healthcare systems, Lebanese nurses have been exposed to additional stressors, including financial instability, shortages of medical supplies, workforce migration, and increased workload. These contextual factors are supported by previous research indicating that resource limitations and economic instability significantly increase occupational stress among healthcare workers (16, 36). These high levels of stress were also combined with a moderate level of satisfaction, where almost half of the sample had an intention to leave their job. This is consistent with Piotrowski et al. (37) which found about one-fourth of the sample indicated having a strong intention to quit the workplace, and a comparable percentage indicated having a low level of work satisfaction. These findings provide cause for alarm. In a similar research, 83.6 percent of the cohort indicated having a moderate or high desire to quit, although assessments using a comparable scale showed that almost half of a sample of Iranian nurses did (38). The moderate level of job satisfaction observed in this study is consistent with findings from previous studies conducted during the COVID-19 pandemic, which reported decreased satisfaction among nurses due to increased workload and psychological strain (39, 40). However, in the Lebanese context, job satisfaction may be further negatively influenced by economic instability, reduced financial incentives, and deteriorating working conditions. These findings align with research suggesting that financial stress and organizational challenges play a critical role in shaping job satisfaction among nurses in crisis settings (20).

In addition, Slovene research evaluating the pre-pandemic to the pandemic periods found that a sample of healthcare workers experienced much more personal work - related burnout symptoms in 2020. It is well established that long-term social and psychological occupational pressures can lead to burnout syndrome. As per the job demands resource’s philosophy, it makes sense that tasks throughout a disease outbreak would be marked by a lack of controllability and increasing pressure from the health industry, which, per the literary works, raises the risk of work overload and has a deleterious influence on the intention to quit, among other factors. The researcher collected data from nurses who represented a workplace that underwent a quick and significant transformation. It included, along with a few other things, adopting new work processes quickly, moving patients and employees, changing the organizational structure and outpatient procedures, and confronting a significant danger of disease and mortality (41).

Consequently, the significant negative association between stigma-related stress and job satisfaction is a critical finding of this study. This relationship may be explained by the social and psychological burden experienced by nurses during the COVID-19 pandemic. In the Lebanese context, stigma may be intensified due to fear of infection, limited public awareness, and concerns about transmitting the virus to family members.

This finding is consistent with previous studies indicating that perceived stigma and social isolation are strongly associated with reduced job satisfaction and increased psychological distress among healthcare workers (16, 31).

From an organizational perspective, stigma may also reflect a lack of institutional support, insufficient communication, and inadequate protective measures, which can negatively influence nurses’ perception of their work environment. Addressing stigma through awareness campaigns, psychological support, and organizational interventions may therefore play a key role in improving job satisfaction. Our results showed that the anxiety relating to stigma of infection was negatively correlated with lower satisfaction with social interactions, professional development, work control, relationships with coworkers, and total satisfaction. This is supported by the research findings of Said & El-Shafei (32) which found that through an assessment of the components related to the factor “relationship with colleagues” among ICU nursing professionals disclosed that the inability to share perspectives and emotions with colleagues was a major workplace source of stress. This could be explained by a lack of opportunities due to the enormous volume of work linked to COVID-19. Additionally, examination of the component “dispute with superiors” showed that important stresses that definitely worsened with the severe workload connected to COVID-19 were the absence of assistance from immediate superiors and the common practice of blame for uncontrolled mistakes. This is in line with the findings of Varghese et al. (42), who claimed that other medical professionals frequently use nursing staff as scapegoats.

Further work-related concerns emerged during the COVID-19 pandemic, with greater vulnerability to the risk of infection ranking as a significant source of stress. All health workers are subject to this hazard, although not all healthcare practitioners are at the same level of risk. Compared to other specializations, some, including emergency and critical care nursing, may carry a higher risk. These nurse practitioners are especially at risk since they frequently attend to COVID clients first and experience a lot of work stress (43). The research participants’ sensitivity to the risk of infection was increased since they were ICU nurses who worked in a hospital setting. Stigmatization, where individuals may view health care workers as mobile sources of contamination in the population, is an additional aspect of the stress arising from the infection hazard (44), hence stigmatization was a substantial source of stress for staff nurses in our research. While using strong biosecurity precautions is necessary to avoid the transmission of illness to oneself and others, it was a significant source of worry for the involved nursing professionals. This is in line with the findings of Akkuş et al. (45), who noted that thermal discomfort, water reduction, and lowered dexterity caused by numerous layers of gloves, adjustable face shields that hinder vision, and backache, were all regarded as major stressor. Interpersonal anxieties and pressures were also important COVID-19-related stresses for the ICU staff nurses in our study. These sources of stress were consistent with earlier studies, which found that individuals’ fears of isolation, spreading the infection to their friends or family members, the unpredictability of the epidemic’s period, the lack of available medical care, headlines of new COVID-19 case scenarios disclosed in the media, and deficiencies of personnel, equipment, and sufficient precautionary equipment were all key challenges. HCWs who endured SARS and MERSA-COV reported experiencing similar stresses, however to varying degrees (46).

This research’s ICU nurses experienced a moderate level of overall job satisfaction due to their very stressful work atmosphere. The association between stress related to stigmatization and work satisfaction was particularly strong. Previous research suggesting that stress at the personal level impacts work satisfaction (40) lends credence to this finding. For example, a prior research by Shen et al. (47) revealed that the primary causes of job discontentment between nurses working in intensive care units were first, being unsatisfied with external incentives of their position, appreciation, and acknowledgement, reflecting their perception that their demanding work was not adequately compensated financially and morally; second, being unsatisfied with timetabling, family and career alignment, and team relationship that was undoubtedly impacted by COVID. Third, the nurses’ capacity for decision-making is hampered by their dissatisfaction with control and responsibility as a result of ongoing revisions to official guidelines and rules related to COVID-19.

Furthermore, the results of our study showed that the satisfaction scores were significantly associated with intent to leave recorded by the ICU nurses. This association between job satisfaction and intention to leave is consistent with existing literature, which suggests that lower levels of job satisfaction are linked to higher turnover intention among nurses. This relationship may reflect the impact of unmet expectations, workload pressures, and limited professional development opportunities.

In the Lebanese context, economic instability and reduced financial incentives may further weaken job satisfaction, thereby increasing the likelihood of nurses considering leaving their positions. Because there is a negative cycle among job satisfaction and stress at work, nurses frequently think about leaving their positions at institutions or even switching careers when they experience high levels of stress (48). This finding may help explain the higher proportion of ICU nurses reporting intention to leave in this study. Previous research during the SARS pandemic in 2003 revealed that HCWs indicated unwillingness to work or elevated resignation thoughts (26).

Finally, for the prediction model, marital status predicted the stress experienced by the nurses and their educational level also predicted their job satisfaction. This is consistent with Piotrowski et al. (37) which have found the educational level and higher years of experience predicted lower stress and higher satisfaction.

Moreover, the finding that age was a significant predictor of intention to leave provides important insight into workforce dynamics. Younger nurses may be more likely to report intention to leave due to greater career mobility, increased opportunities to work abroad, and dissatisfaction with local working conditions. In contrast, older nurses may demonstrate greater job stability due to family responsibilities, financial commitments, and longer tenure within the organization. This interpretation is supported by previous studies showing that younger healthcare workers are more likely to consider leaving their positions, particularly in high-stress environments (36, 49).

It is important to note that the cross-sectional correlational design used in this study limits the ability to establish causal relationships between occupational stress, job satisfaction, and intention to leave. Therefore, the findings should be interpreted as associations rather than cause-and-effect relationships. Future longitudinal or experimental studies are recommended to better understand causal pathways.

The findings of this study highlight the importance of contextualizing occupational stress and job satisfaction within the broader socio-economic environment. In Lebanon, the ongoing economic crisis, currency devaluation, and healthcare workforce migration have created additional burdens on nurses. Previous literature has emphasized that such systemic stressors can exacerbate burnout and turnover intention among healthcare professionals (36, 49). Therefore, the elevated stress levels and moderate satisfaction observed in this study may reflect not only the impact of the COVID-19 pandemic but also the cumulative effect of long-standing structural challenges within the Lebanese healthcare system.

## Conclusion and recommendations

This study identified significant associations between occupational stress, job satisfaction, and intention to leave among critical care nurses during the COVID-19 pandemic. These findings have important implications for healthcare administrators and policymakers, particularly in resource-constrained settings such as Lebanon.

To address occupational stress, healthcare institutions should implement structured mental health support programs, including access to counseling services, stress management training, and peer support systems. In addition, organizational efforts should focus on reducing stigma through awareness campaigns, open communication, and supportive leadership practices.

Improving job satisfaction requires targeted strategies such as enhancing financial incentives, ensuring fair workload distribution, and providing opportunities for professional development and career advancement. Given that younger nurses were more likely to report intention to leave, retention strategies should specifically address their needs by offering career progression pathways and improving working conditions.

At the policy level, national healthcare authorities should consider developing workforce retention plans that address nurse migration, resource allocation, and long-term system resilience. These context-specific and actionable strategies are essential to support nurse well-being and ensure the sustainability of the healthcare workforce in Lebanon.

## Data Availability

The raw data supporting the conclusions of this article will be made available by the authors, without undue reservation.
