# Correction: Occupational stress, job satisfaction, and intention to leave among critical care nurses during the COVID-19 pandemic: a cross-sectional study in Lebanon

**DOI:** 10.3389/fpubh.2026.1901548

**Published:** 2026-07-20

**Authors:** Manar Bani-Hani, Suhair Al-Ghabeesh, Ahmad Rayan, Patricia Tai, Mirna Fawaz, Zeina Salloum, Hanan Alyami

**Affiliations:** 1 Faculty of Nursing, Al-Zaytoonah University of Jordan, Amman, Jordan; 2 Faculty of Nursing, Zarqa University, Zarqa, Jordan; 3 Department of Oncology, University of Saskatchewan, Saskatoon, SK, Canada; 4 Faculty of Health Sciences, Nursing Department, Beirut Arab University, Beirut, Lebanon; 5 Department of Medical and Surgical Nursing, College of Nursing, Princess Nourah bint Abdulrahman University, Riyadh, Saudi Arabia

**Keywords:** critical care nurses, intent to leave, COVID-19, occupational stress, job satisfaction, public mental health

The funder Princess Nourah bint Abdulrahman University Researchers Supporting Project number (PNURSP2026R386), Princess Nourah bint Abdulrahman University, Riyadh, Saudi Arabia and the Deanship of Scientific Research at Zarqa University, Jordan were erroneously omitted.

The correct **Funding** statement appears below:

The author(s) declared that financial support was received for this work and/or its publication. This study was supported by Princess Nourah bint Abdulrahman University Researchers Supporting Project number (PNURSP2026R386), Princess Nourah bint Abdulrahman University, Riyadh, Saudi Arabia. The research received funding from the Deanship of Scientific Research at Zarqa University, Jordan.

In the published article, the “Acknowledgments” section was erroneously omitted.

The correct **Acknowledgments** appears below:

“The authors acknowledge the support of Princess Nourah bint Abdulrahman University through the Researchers Supporting Project (PNURSP2026R386), Riyadh, Saudi Arabia. The authors also acknowledge the Deanship of Scientific Research at Zarqa University, Jordan, for their support.”

The original version of this article has been updated.

